# Editorial Note: Novel randomization and iterative based algorithms for the transactions assignment in blockchain problem

**DOI:** 10.1371/journal.pone.0349918

**Published:** 2026-05-26

**Authors:** The PLOS One Editors

The *PLOS One* Editors issue this Editorial Note because this article [1] was identified as one of a series of submissions for which we have concerns about aspects of the peer review process. Readers are advised to interpret the article [1] with caution.
